# Exploring young people's perspectives on mental health support: A qualitative study across three geographical areas in England, UK

**DOI:** 10.1111/hsc.14078

**Published:** 2022-10-25

**Authors:** Eleanor Holding, Mary Crowder, Nicholas Woodrow, Naomi Griffin, Nicky Knights, Elizabeth Goyder, Rachael McKeown, Hannah Fairbrother

**Affiliations:** ^1^ School of Health and Related Research (ScHARR) University of Sheffield Sheffield UK; ^2^ Population Health Sciences Institute Newcastle University Newcastle UK; ^3^ Social Sciences, Cavendish Campus University of Westminster London UK; ^4^ The Association for Young People's Health London UK; ^5^ Health Sciences School University of Sheffield Sheffield UK

**Keywords:** inequalities, mental health, mental health support, young people, youth work

## Abstract

Improving young people's (YP) mental health and well‐being is a global public health priority. Despite continued commitment within the UK policy agenda to improve the mental health and well‐being of YP, the incidence of mental health issues continues to rise. This has been further compounded by the outbreak of COVID‐19 which has disproportionately affected YP in the most socioeconomically disadvantaged areas. Understanding YP's perspectives on what supports their mental health is important to develop policies that meet their needs. We conducted focus groups (*n* = 18 with 42 YP aged 13–21) in three geographical areas with high levels of deprivation in England, UK. Recruited through six local youth organisations, each group of YP took part in three interlinked focus groups designed to explore their perceptions of what impacts their health in their local area, and their understandings of health inequalities through participatory methods. Throughout their discussions, YP foregrounded the significance of mental health and mental health support structures. YP perceived challenges to accessing mental health provision and an unmet need for support within their local communities. Alongside this, YP consistently highlighted the importance of youth groups for promoting good mental health and mitigating challenges to poor mental health. However, ongoing cuts to the voluntary sector and universal services continue to impact areas and individuals in the greatest need. In the face of deficits in formal mental health support, our findings highlight the pressing need for increased investment in services focused on prevention (such as youth groups) in areas of high deprivation.

**What is known about the topic?**

- There is an increasing prevalence of mental health issues in young people globally, exacerbated by the COVID‐19 pandemic.
- There are many challenges in accessing mental health support for young people.

**What does this paper adds?**

- Young people are acutely aware of issues within their local areas, such as low income and poverty, and how this impacts on mental health.
- Young people perceive significant shortcomings in current provision both in terms of promoting positive mental health and providing support for mental health challenges.
- Young people foreground the importance of youth organisations in supporting positive mental health. Such findings provide insights into the forms of support that may be most accessible and acceptable to YP in deprived communities.

## 1 INTRODUCTION

Improving young people's (YP) mental health and well‐being is a global public health priority (Kieling et al., 2011; UNICEF, 2021). Mental health disorders constitute a major burden of disease for YP globally, with estimates suggesting that 13% of adolescents aged 15–19 have a diagnosed mental health condition (UNICEF, 2021), increasing to approximately 16% aged 5–16 in the United Kingdom (NHS Digital, 2020). Further, globally we have seen increasing inequalities in mental health outcomes (Patel et al., 2018), with prevalence strongly associated with several key sociodemographic characteristics: such as age, sex, ethnicity, sexuality and socioeconomic position (Deighton et al., 2019; Ferrari et al., 2013; Gutman et al., 2016; Semlyen et al., 2016). For example, in the United Kingdom the prevalence of mental health problems is four times greater among 11‐year‐olds in the most deprived quintile compared to those in the least deprived quintile (Gutman et al., 2016). These issues have been further compounded by the COVID‐19 pandemic which has exacerbated poor mental health for the already most disadvantaged YP (Patel et al., 2020; YoungMinds, 2020). Indeed, lockdown and mandated social distancing measures, home‐schooling, increased isolation/loneliness and service closures reducing access to informal and formal support have been noted to have had detrimental impacts on YP's mental health (Mind, 2021; Singh et al., 2020; YoungMinds, 2020).

In light of increasing rates of mental ill‐health among YP and the wider impacts of the COVID‐19 pandemic, the State of the World's Children report (2021) notes the pressing need to take global policy action to promote good mental health for all YP (UNICEF, 2021). However, approaches tend to focus on improving support within formal health and educational settings (e.g. Department of Health & Social Care & Department of Education, 2018). In the United Kingdom, a 2018 Green Paper on YP's mental health set out measures to improve and embed mental health support within schools and colleges by introducing a designated lead for mental health support, as well as to fund new mental health support teams, and pilot a four‐week waiting time for access to specialist support (Department of Health and Social Care & Department of Education, 2018). The NHS Long Term Plan (NHS England, 2019) also proposed new measures such as funding community‐based mental health services for YP. However, such policies have been critiqued for focusing on joined‐up working rather than increasing service capacity (Griffin et al., 2022). This increased policy attention is set against the context of already strained specialist service provision due to continued funding cuts (Edbrooke‐Childs & Deighton, 2020; Harris et al., 2019). Funding for child and adolescent mental health services (CAMHS) has stagnated, with local Clinical Commissioning Groups, on average, spending under 1 per cent of their budgets on YP's mental health whilst public mental health services, school nurses and other community mental health support have seen a £700 million reduction in funding between 2014/15–2020/21 (Local Government Association, 2022). This systemic underfunding of mental health services has left providers unable to meet increasing demand including ‘record high’ referrals to CAMHS (NHS Digital, 2021; Trades Union Congress, 2018). Many YP wait months for treatment (Crenna‐Jennings & Hutchinson, 2020), with 1 in 5 waiting for 6 months or longer (NHS Digital, 2018).

There are many barriers around accessing mental health support for YP such as complicated referral processes, long waiting times, high support thresholds from statutory services and a lack of available provision (Crenna‐Jennings & Hutchinson, 2020; Edbrooke‐Childs & Deighton, 2020; Moore & Gammie, 2018; Radez et al., 2020). Beyond service level and resourcing‐related barriers, the perceived stigma and embarrassment associated with accessing formal support, a lack of trust in professional confidentiality (Radez et al., 2020) and a preference for alternative, and often informal, support avenues, can all impact on YP receiving appropriate support (Chan & Quinn, 2012; Gulliver et al., 2010). Thus, resourcing support without improving accessibility and acceptability for YP may be a redundant approach. In light of these issues, it is vital to explore the perspectives and experiences of YP around mental health support and service provision so that policies can be developed which better meet their needs.

### 1.1 Study aim

Drawing on data from a wider study exploring YP perceptions of factors affecting health in their local area and understandings of health inequalities (Fairbrother et al., 2022), this paper aims to present YP's views and experiences of mental health issues and support to explore how mental health provision could be better tailored to their needs.

## 2 METHODS

We recruited six groups of YP (*n* = 42) aged 13–21 from youth groups across three areas in England (South Yorkshire (SY), the North East (NE) and London (L)). All three areas fell within the most deprived quintile based on the 2019 English indices of multiple deprivations (IMDs). We undertook a series of three interlinked qualitative focus group discussions with each group, resulting in 18 focus groups in total. The majority of focus groups were conducted online (*n* = 15) due to COVID restrictions. Focus group discussions (*n* = 3) with one youth group were conducted face‐to‐face when restrictions were lifted. Data collection occurred between February 2021 and June 2021. Ethical approval for the study was granted by the School of Health and Related Research (ScHARR) ethics committee at the University of Sheffield (ethics form reference number: 037145).

### 2.1 Participants and recruitment

YP were recruited through local youth organisations. Youth workers supported recruitment by disseminating brief project information sheets and informal descriptions of the project. Written consent was obtained for all participants, with opt‐in consent from parents/guardians for those under 16. Our final sample consisted of 42 YP aged 13–21. Participants were asked to provide basic demographic information, but this was anonymised at the point of collection to protect confidentiality, and to minimise potential identification (see Table 1 for an overview of participant demographic information).

**TABLE 1:** Participant demographics

Number of participants	Age	Gender	Ethnicity
42	Age range: 13–21 Average age: 16.7	18 Female 19 Male 2 Non‐binary 2 Trans Male 1 Gender Fluid	30 White British 6 Asian/Asian British 3 Black/Black British 2 Mixed/Multiple ethnic groups 1 Chinese

### 2.2 Data generation

Data was generated through focus group discussions. Each discussion lasted between 90 and 100 min. The focus groups employed a semi‐structured approach to discuss perceptions of factors influencing YP's mental and physical health and opportunities to be healthy in their local area.

Due to the ethical considerations of confidentiality in focus groups (Bryman, 2016), and the potentially sensitive nature of discussing topics related to health and inequality (Martins et al., 2018), we gave considerable attention to the data collection methods and tools. We had support facilitating the focus groups from partnering youth workers, providing benefits to both engagement (facilitating participation) and protection (when discussing potentially sensitive topics). Details of the methodological and ethical challenges encountered in this study and how they were overcome, as well as the benefits of working with youth organisations to facilitate data collection, have been reported in a previous paper (Woodrow et al., 2022).

### 2.3 Data analysis

The focus groups were audio‐recorded with consent from the participants and parents/guardians (where applicable). Recordings were transcribed verbatim by third‐party transcription companies and were anonymised at the point of transcription. Transcripts were analysed using thematic analysis, informed by Braun and Clarke's (2006) framework. Data were coded within NVivo‐12 software using a coding framework developed by the research team (Fairbrother et al., 2022). This framework provided an initial scaffolding to guide analysis, but specific themes were developed from close reading of the transcripts.

We present verbatim quotes and conversation extracts from the focus group discussions to highlight the key themes which we generated through analysis. Participant quotes are accompanied with the field site focus group session only (e.g. SY, 1.1, NE, 2.3). To minimise participant identification, no further demographic or characteristic information is presented, but we make reference to this in the findings if important.

## 3 FINDINGS

Four key themes arose from our analyses: (1) factors influencing YP's mental health, (2) deficits in mental health provision: the importance of prevention, (3) benefits and challenges of mental health support in schools and (4) the importance of youth groups for mental health.

### 3.1 Factors influencing YP's mental health

During discussions around factors influencing health, YP often foregrounded issues relating to mental health and well‐being. YP described how mental health issues could be experienced by all people, regardless of their personal circumstances.

“I don't think people sometimes realise how much mental health can affect young people, you know, you get mental health in like every single age, from teenagers to elderly people.” (SY, 2.2)

YP demonstrated nuanced understandings of the factors impacting opportunities to enjoy good mental health. It was clear that the issues faced by YP were complex and wide‐ranging including: social anxiety, exam stress, peer pressure, bullying, fear due to perceptions of safety, low self‐esteem and body image. Although such issues could be experienced by all YP, participants in this study demonstrated an acute awareness of how wider sociodemographic factors such as living in areas of high deprivation/poverty, ethnicity, sexual orientation/gender and intersecting disadvantages, shaped mental health outcomes. For example, YP in London (whose youth groups were much more ethnically diverse than the other groups) highlighted how people from minority backgrounds were more likely to experience poor mental health than White British people:

“I think inequalities often disproportionately affect minorities and I think if you're in a minority group it can also be quite isolating which can also influence, like, the stress you experience, not just because of how people treat you but because, because you're isolated from other people.” (L, 2.2)

YP also highlighted issues which they perceived to be particularly pertinent within their local areas. YP demonstrated a clear perception of how factors, such as lack of access to services/activities, food (in)security, home/family relationships and crime rates and safety in the local area were influenced by socioeconomic position. They particularly highlighted the impact of living in poverty on chronic stress:

“People from lower income backgrounds are more likely to be more stressed because often they've got children to care for whereas people from higher income backgrounds can afford childcare as well. And also the stress of not having enough money can like really affect them mentally so they're more likely to get mental health problems and the suicide rates are probably much higher for people from lower income backgrounds.” (SY, 2.2)

In turn, there were discussions of how COVID‐19 had exacerbated many issues for all YP but particularly for those living in poverty. For example, YP talked about how factors that promote good mental health, such as the ability to socialise and speak to friends or exercise was harder for people in poverty due to digitial divides and social distancing. A lack of access to personal outdoor/green space such as a garden and high crime rates in the local area prohibiting safe access to public space contributed to poor mental health: “So obviously some people might be in a small flat or whatever, no garden, they might not have the space to exercise either indoors or outdoors as such.” (SY, 2.2)

### 3.2 Deficits in mental health provision: the importance of prevention

YP discussed the importance of universal access to mental health services, and frequently highlighted deficits in mental health provision. In particular, they described a lack of access and availability of statutory services and long waiting lists for mental health support such as CAMHS*^1^: “you've got to wait like two years for CAMHS or something like that. I've been waiting like three or four years to get onto it” (SY, 2.2).

YP consistently asserted that mental health support should be accessible and available to everyone who needs it, irrespective of the severity of the mental health problem. However, several YP perceived that the threshold for specialist support was too high, meaning that only those who had reached ‘crisis point’ could access services: “I think that the people who aren't at risk then only get help when they deteriorate…it's like there's a standard for how bad mental health has to be for you to get help” (SY, 2.3). This contrasted with their emphasis on the importance of early intervention and support to prevent problems escalating: “[CAMHS] should put out sparks instead of fires and focus on problems early on. They just tend to focus on crisis when things are at the extreme” (NE, 1.2).

Further, some participants described how pressure on services was impacting upon quality of care. When YP were able to access CAMHS support, some discussed deficits in staff knowledge, competence and allocated support time, leading to negative experiences of the service. YP described how high staff turnover with therapists and care co‐ordinators frequently leaving or changing was a common issue which exacerbated pre‐existing mental health issues and made it difficult for YP to build rapport with health professionals:

“you're dropping a worker and then you're going back to one and then you're getting a new one like every week, basically. And it's not like because you're building that stuff again and then they're going and then that puts like more anxiety in everything. So you have more problems to worry about.” (NE, 1.2)

In turn, YP also described treatment feeling rushed and being discharged from services before feeling ready:

“to me it feels like they push their kids away, like once they're smiling they think ‘oh yeah, my job's done here’ and then they can go and help another kid.” (SY, 1.3)

Despite some negative views, YP were acutely aware of the external factors impacting the delivery of care. They acknowledged that working in CAMHS would be a challenging job, and that staff turnover may be due to stress, as well as a lack of funding and resource. Participants also advocated for greater resource allocation for training staff to appropriately support the broad range of mental health issues experienced by YP:

“Since [CAMHS] is heavily unfunded and a lot of staff don't really know what they're doing, essentially, because they haven't got the experience and the proper training since it's been unfunded, not that well…a lot of the time, with CAMHS and stuff, for depression, they'll say, ‘Are you feeling suicidal? Have you self‐harmed? If not, then you're not depressed’.” (NE, 1.3)

Some YP discussed the impact of reduced funding on the delivery of services and how this varied by geography. For example, some YP described variations in service provision between the north and the south, as well as between smaller areas within their local communities:

“like where I live it is, well I feel like there's not enough being done for equal like equipment and things like that for our mental health as well, for young people as well. I feel like we have to shout and scream in order to get heard, it's not just done because they think about us, whereas I feel like in comparison to other boroughs they tend to do it for their younger people” (L, 1.1)

### 3.3 Benefits and challenges to mental health support in schools

Schools were seen as potentially valuable places to deliver mental health support, as staff have the ability to identify and respond to issues as they arise. In some cases, teachers and school staff were seen as “trusted” sources of support. This was particularly evident when there were dedicated staff members for support:

“I think what I found helpful at my school was having specific people that you were told you could go to and actually, like, seeing their faces and not just them being in a random office, somewhere, like, you kind of, did get to interact with them.” (L, 2.3)

However, it was acknowledged that staff members in schools did not always have the capacity or knowledge to identify and appropriately support mental health problems alongside their day‐to‐day roles.

“Teachers have so much to do and you also don't want to like put too much on the teacher because they've got whole year groups to manage.” (L, 1.2)

Echoing earlier discussions around the risk‐based targeting of mental health support from specialist services (see above), some YP felt that support in schools was only offered at crisis points:

“one of my friends, she used to go to wellbeing a lot trying to get support and it was like ‘oh no, it's not that bad, you're exaggerating’ and then she attempted [suicide] – and then after that was like constantly with her 24/7 and again, someone went again saying their mental health was bad, ‘oh no, you're exaggerating’. It's like they only really listen to you when it's gone that far.” (SY, 1.2)

Where support is available in school, YP discussed how there was a lack of longer‐term follow‐up once there was a sign of improvement, leaving them to potentially deteriorate without someone checking‐in. Confidentiality and trust were also raised, with some YP discussing previous experiences of mental health professionals sharing private conversations with teachers without consent. This meant they were less likely to consider school staff as a viable source of support:

“but like I wouldn't feel comfortable going to a member of school because I feel like in my past experiences with like counsellors and people in school, they've gone and spoken about things that I've told them to other teachers.” (SY, 1.3)

More generally, YP also perceived a lack of education, support and understanding around more complex mental health problems within schools. YP described education on generic mental health issues (e.g. anxiety and depression) but this was seen as superficial and out of touch with everyday experiences. YP described a lack of education on important topics such as eating disorders or gender transitioning:

“In my school the teachers talk about it, they do things like awareness days and PowerPoints, but they kind of set it out as ‘you can have this type of depression, you can have this type of anxiety or you can have this’. As soon as you come to them with something different they're like ‘are you sure though? Is that really a thing? Is there anything we can actually do about that?’” (SY, 1.3)

Echoing previous discussions around specialist mental health service provision, YP stated that more funding, resource and staff training was required to ameliorate current issues around mental health provision and support in schools.

### 3.4 The importance of youth groups for mental health

In contrast to the often negative discussion of mental health support from specialist services and schools, YP frequently described how youth groups supported their mental health. They talked about how youth groups provided them with a “safe” space to discuss their problems and “escape” potential issues at home:

“[name of youth organisation], it's a place where we all feel safe, a place where other young people have a place where we can speak freely and be ourselves.’” (SY, 2.2)

They also highlighted how youth groups afforded the opportunity to socialise and “bond” with likeminded YP facing similar issues, instilling a general sense of belonging and empowerment. This provided a great source of peer support for participants:

“there's the [name of youth organisation] and that's where me, [name] – my other mate – and [name] go on a Wednesday…they help me a lot with stuff that's going on and it helps because like all me mates are there and it's just a lot easier for me to see them.” (SY, 2.1)

“[name of youth organisation] is good for our health because we hang out with friends and we play a lot in the back.” (NE, 2.1)

Participants from particularly marginalised groups, such as LGBTQ young people, described how this form of peer support was especially important for them as they often did not feel safe in other spaces. YP stated that being around YP with similar experiences who “know what we are going through” (NE, 2.1) provided an important source of emotional support.

In this way, it was clear that youth groups promoted positive mental health: “So things like youth clubs, which are like [name of organisation], help us to basically have, improve on our own social and mental health” (L, 1.1).

Youth groups also provided connection and referral to onward services and health‐promoting activities. Youth workers were described as providing ancillary support to YP, acting as a non‐specialised, but robust contact for support over a range of issues. It was clear YP felt ‘safe’ to discuss their problems with their youth workers without judgement: “I would say [name of youth group] helps my health…Because I feel safe and I can tell them stuff that I need to. They're very very supportive” (SY, 2.1).

Thus, youth workers were considered important sources of emotional support, with one participant describing them as like a “therapist”:

“You can go in, speak to a youth worker and actually get your thoughts onto paper and actually help you, as if they're a therapist. They can actually help you, and I've gone through that with youth workers, and they've helped me with that.” (L, 1.1)

A crucial and consistently highlighted benefit of youth clubs was that they were accessible and affordable for those who needed them. Indeed, whilst YP described numerous health‐promoting spaces and activities (e.g. sports clubs), they acknowledged several barriers to participation. YP recognised health‐promoting spaces as places where they could socialise/exercise, feel safe and engage in activities that support their physical and mental well‐being, but they saw these as being inaccessible for many YP. The high cost of some activities (e.g. gym membership), a lack of advertising to understand what is available and issues with the costs and availability of transport were notable barriers to participation. Improving access and availability through increases in funding and resource for youth groups was consistently foregrounded as a key priority by YP. Indeed, they saw youth organisations as both integral but underappreciated:

“More money needs to be provided because like they need to get paid for actually – I know they do it apparently to just help kids and stuff like that, but it's like they're taking it out of their free time and they're choosing to do that instead of spending time with their family. I just feel like there needs to be more money provided for both the workers but also for the youth club.” (SY, 2.2)

## 4 DISCUSSION

This study aimed to explore YP's understandings of what influences their opportunities to be healthy within their local area and their understandings of health inequalities. Through their narratives, YP foregrounded mental health provision as an important factor which influences their health. Our findings echo previous research into deficits in mental health support within healthcare services and school settings (e.g. Care Quality Commission, 2017, Creswell et al., 2021, see below). However, a significant finding is that YP were acutely aware of how poverty impacted mental health and how funding cuts impacted on the availability of support within their local areas. In particular, they perceived challenges and unmet support needs within local service provision, and clearly identified what could help from their experiences in youth groups. Our findings provide insights into forms of support that may be most effective and appropriate for YP in deprived communities. In particular, they demonstrate the need for mental health support which is accessible, affordable, tailored to the needs of YP and delivered by trusted adults based in their own communities.

YP were aware of how funding cuts and gaps in provision directly impacted on their everyday lives, with long waiting times and high thresholds for mental health support (Care Quality Commission, 2017), high turnover of therapists due to stress levels, (Creswell et al., 2021; Owens & Charles, 2016) early discharge from care (Owens & Charles, 2016), poor resources and low pay for youth organisations/youth workers, and the need for more capacity, staff training and support within schools and health service settings. Such findings are significant given the push to deliver the ‘levelling up’ agenda in the United Kingdom which calls to reduce inequalities in health between the richest and the poorest (Department for Levelling Up, Housing and Communities, 2022). Despite commitments to reduce inequalities, our study highlights how cuts to services are experienced by YP living in areas of high deprivation. In the context of ever‐increasing socioeconomic and health inequalities (Marmot et al., 2020), it is crucial to explore and maximise the benefits of alternative avenues of support for YP, as well as improving the accessibility and acceptability of existing mental health provision in areas of the greatest need.

While schools may be well positioned to identify mental health problems (Fregredo‐Childs et al., 2020), previous studies have noted issues in staff feeling ill‐equipped to identify potential issues (Soneson et al., 2020), leading to staff missing or misidentifying YP's mental health needs (Anderson et al., 2019). Practitioners have also highlighted the limited capacity that school staff have for mental health support, and the potential exclusion of YP not in formal education, training or employment (NEETS) in school‐centred approaches (Griffin et al., 2022). Similarly, although some YP in our study felt that schools were a good place to receive mental health support, several felt staff lacked the time, training and knowledge to provide mental health support, especially for more complex mental health problems or for YP who required early intervention. This adds support to the introduction of designated mental health leads in schools (Department of Health and Social Care & Department of Education, 2018), but highlights how staff must have robust knowledge of a range of issues YP can experience. This calls for further training, funding and resource to strengthen and widen mental health support within schools. In particular, our findings point towards the need for specialised staff members dedicated to mental health support (Jessiman et al., 2022).

A key finding of our study is the importance YP placed on youth groups as a support system for their mental health and well‐being. As well as providing safe, easily accessible, informal and affordable support on an ongoing basis, youth workers provided an important liaison function between YP and other forms of potential support. Being in informal settings around like‐minded YP facing similar issues also provided an important source of peer support which is not always available from other forms of individualised health service provision. For many of the YP in our study, their youth organisation plugged gaps in existing mental health service provision, providing support in times of increased challenges due to COVID‐19. Indeed, previous studies have highlighted the beneficial role of youth work in fostering social connections and ‘safe spaces’ (Coburn, 2011; Fish, 2014; Fyfe et al., 2018), which can encourage and facilitate support‐seeking for YP (Gondek et al., 2016; Gulliver et al., 2010).

### 4.1 Implications for policy and practice

Public service cuts have disproportionately affected the poorest and most vulnerable (Cummins, 2018; Hastings et al., 2015). This has resulted in increasing inequalities in the distribution of youth services across the country (YMCA, 2020), with YP in more affluent areas being twice as likely to have access to youth services than their poorer counterparts (Booth, 2021). Through their narratives on the impact of poverty and the challenges faced by providers, YP in our study showed how they have acute awareness of and directly experience the impact of austerity policies. Given current deficits in mental health service provision and inequalities in mental health for those in the poorest areas (Care Quality Commission, 2017), and local authority funding for youth services in England (with a 71% reduction since 2010) (YMCA, 2020), there is a clear need for investment in services which focus on supporting mental health through prevention (such as youth services) in areas of the greatest need (Department of Health & Social Care, 2022). Our findings add weight to recommendations made in the 10‐year mental health and well‐being plan consultation for the pressing need to focus on prevention and reduce mental health disparities (Department of Health & Social Care, 2022).

In turn, our findings add to the importance of reducing the waiting times YP experience when waiting for mental health support. This requires renewed investment and ring‐fenced funding for CAMHS services (Royal College of Paediatrics and Child Health, 2020), full implementation of the NHS Long Term Plan commitments and expansion of the NHS CYPMH waiting time pilots (NHS England, 2022). However, our study has shown that it may not be enough to invest in services without improving existing provision. Alongside increased investment services need to be accessible and acceptable to YP. Through exploring YP's perceived challenges in accessing mental health support this study provides important insights into how services can be designed to better meet their needs.

### 4.2 Strengths and limitations

Exploring YP's perspectives on health is a clear strength of this study given the paucity of previous research exploring the views and experiences of YP (Smith & Anderson, 2018). Although waiting lists and the availability of mental health service provision vary by geography and thus our findings may not represent the views of all YP, they do highlight important issues which are pertinent across different geographical areas. Further, undertaking the study during COVID‐19 allowed for interesting reflections due to a potentially heightened awareness among YP on the importance of mental health. Our open approach to exploring YP's perspectives on what influenced their health resulted in YP prioritising mental health and service provision in their narratives.

Recruiting through youth groups afforded many benefits to the project (see Fairbrother et al., 2022; Woodrow et al., 2022). Youth workers helped facilitate participant engagement and provided a trusted source of support to participants (Woodrow et al., 2022). However, the presence of youth workers throughout the recruitment and data collection process may have also influenced YP's discussions on youth groups in a positive direction. It therefore may not be surprising that YP in our study emphasised the importance of youth work given their regular involvement with youth organisations. Nevertheless, the YP we worked with offered clear examples of how youth groups in areas of high deprivation specifically supported YP's mental health as well as providing insights into the challenges and unmet need for mental health support within their own communities. Such findings emerged from youth‐led discussions without prompting from the research team or youth workers. Our research provides wider/more generalisable insights into the forms of support that may be most accessible and acceptable to YP in deprived communities.

## 5 CONCLUSION

YP are acutely aware of the impact of poverty and cuts to local services on their mental health and the availability and accessibility of mental health support. In the context of an increasing emphasis on reducing health inequalities, our study supports calls to reduce geographical inequalities for those in the greatest need (Marmot et al., 2020). YP consistently foreground youth organisations as important promoters of positive mental health, particularly in areas of high deprivation and in the context of cuts to specialist mental health service provision. Through their own experiences with youth groups, YP identified what could help to support mental health. In particular, they identified the need for support which was accessible, affordable and tailored to their needs. Our study supports calls for further investment in preventative services that provide this form of community and peer support, particularly in more deprived communities where the needs are greatest.

## AUTHOR CONTRIBUTIONS

HF and EG conceptualised the study. EH, MC, NW and NG collected and analysed the data. EH, MC and NW wrote the initial manuscript. All authors commented on and edited the manuscript. EH wrote the final manuscript.

## FUNDING INFORMATION

This project is funded by the National Institute for Health and Care Research (NIHR) School for Public Health Research (SPHR) (grant reference PD‐SPH‐2015). The views expressed are those of the authors and not necessarily those of the NIHR or the Department of Health and Social Care.

## CONFLICT OF INTEREST

The authors declare no conflict of interest.

## Data Availability

The data that support the findings of this study are available on reasonable request from the corresponding author. The data are not publicly available due to privacy and ethical reasons.

## Endnote

^1^
Children and young people's mental health services (CAMHS) is used as a term for all services that work with children and young people who have difficulties with their mental health or wellbeing in the UK.
